# The misdiagnose of psychosis and its implications in some cultures. The experience of Doctors Without Borders (MSF) in South Sudan and Chad

**DOI:** 10.1192/j.eurpsy.2025.1286

**Published:** 2025-08-26

**Authors:** C. Carreño Glaria

**Affiliations:** 1 Medecins Sans Frontièrs, Barcelona, Spain

## Abstract

**Introduction:**

In certain cultures psychological suffering is expressed with more somatic symptoms. Besides, they tend to describe more often hallucinations and other forms of perception problems.

**Objectives:**

To describe and warn about the risks of misdiagnose when culture is not an element considered in the diagnosis.

**Methods:**

Descriptive methodology based on practice and observation.

**Results:**

In the last year MSF has been working in more than 40 countries such as South Sudan and Chad with local and refugee populations. The teams have provided treatment to people with anxiety, trauma related and depressive disorders. However, we have also treated many people with hallucinations in the context of trauma, forced displacement. Several patients presented sudden, abrupt symptoms such as fear, insomnia, anxiety, and hallucinations. These hallucinations are often related to the potential traumatic event, to the witness of violence or displacement. When it appears accompanied by flashbacks, hypervigilance, and other PTSD symptoms it may be considered as PTSD with psychotic like symptoms. But other times these hallucinations seem to be more linked to a depressive episode or adjustment disorder. We have witness several cultures where hallucinations are very common during grief processes. The main risks of a misdiagnose of psychosis is the mistreatment.

On another note, it is known that migrant and refugee status are associated with a higher prevalence of psychosis. Using the cultural lenses, should we question these findings? Is there maybe a bias or misdiagnosis in some of these research?

**Conclusions:**

Not all people with sensory perceptual alterations can be diagnosed with psychosis. A proper diagnose that is adapted to the culture of the person is essential for a good quality treatment.

**Disclosure of Interest:**

None Declared

